# Severe Pulmonary Hypertension Meets Intraperitoneal Surgery: No Place to Inflate?

**DOI:** 10.7759/cureus.35318

**Published:** 2023-02-22

**Authors:** Thomas Hickey, Sachidhanand Jayakumar, Albert C Perrino

**Affiliations:** 1 Anesthesiology, Yale School of Medicine, New Haven, USA; 2 Anesthesia, Veterans Affairs (VA) Connecticut Healthcare System, West Haven, USA

**Keywords:** open and laparoscopic surgery, right heart failure, comorbid obesity, pathophysiology of pneumoperitoneum, severe pulmonary arterial hypertension

## Abstract

Severe pulmonary hypertension (PH) is associated with poor operative outcomes; however, guidance for perioperative management of this population is lacking. Mechanical ventilation has known deleterious effects on right ventricular preload and cardiac output. Meanwhile, pneumoperitoneum results in further cardiopulmonary insults. We report the successful case management of a patient with severe PH scheduled for elective cholecystectomy. While patients undergoing this surgery typically benefit from the less invasive, laparoscopic approach, the risk-benefit ratio may tilt towards risk in the setting of severe PH. A multidisciplinary approach to optimize outcome included the decision to perform an open rather than laparoscopic procedure, which resulted in a favorable outcome.

## Introduction

Severe pulmonary hypertension (PH) is associated with poor operative outcomes, and guidance for perioperative management of this high-risk population is lacking [1,2,3,4]. Mechanical ventilation deleteriously affects right ventricular (RV) preload and cardiac output [5]. Pneumoperitoneum adds additional cardiopulmonary strain [6]. Herein we present a multidisciplinary management strategy for such cases, including consideration of performing open surgery to avoid the harms of pneumoperitoneum.

## Case presentation

A 74-year-old man (consent acquired) with recurrent right heart failure with RV systolic pressure >80 mmHg, preserved left ventricular ejection fraction with diastolic dysfunction, previous lower extremity deep venous thrombosis and pulmonary embolism no longer on anticoagulation due to recurrent falls, coronary artery disease, untreated severe sleep apnea (apnea-hypopnea index of 96.5), chronic obstructive pulmonary disease on supplemental oxygen, and BMI of 38 kg/m2, chronic kidney disease (estimated glomerular filtration rate 46 mL/min) presented for cholecystectomy in the setting of chronic cholecystitis after multiple failed therapies over the course of a year including percutaneous drainage and endoscopic retrograde cholangiopancreatography.

American Society of Anesthesiologists (ASA) physical status classification was IV with metabolic equivalents less than four. He was able to transfer from his wheelchair to his chair at home but was unable to walk more than several feet due to joint pain, neuropathy, and dyspnea. The revised cardiac risk index was four, and New York Heart Association functional classification was three. His pulmonary hypertension was thought to be multifactorial, with at least components of WHO group two (left heart disease), three (hypoxemic lung disease), and four (pulmonary arterial obstruction).

Cardiology and pulmonary consultants familiar with the patient emphasized the high-risk nature of the surgery and felt that no further evaluation or changes in medical management were necessary. The anesthesiologist and surgeon similarly described an approximately 20% risk of perioperative death (the NSQIP risk calculator provided a 15.8% risk of death, which was felt to underestimate actual risk due to uncaptured severe comorbidities such as PH); the patient insisted on proceeding. The same anesthesiologist and surgeon then developed a plan addressing patient comorbidities that included modifying the usual surgical approach. Whereas 90% of cholecystectomies at our institution are performed laparoscopically, an open procedure was elected in this case, given the possibly catastrophic pathophysiologic effects of pneumoperitoneum.

On the morning of surgery, a T 7/8 thoracic epidural and radial arterial catheter were placed under local anesthetic. A 3 milliliter (mL) epidural test dose of 1.5% lidocaine with 1:200,000 epinephrine was negative for signs and symptoms of intravascular or intrathecal injection, resulting in an appropriate sensory level to ice.

**Figure 1:**
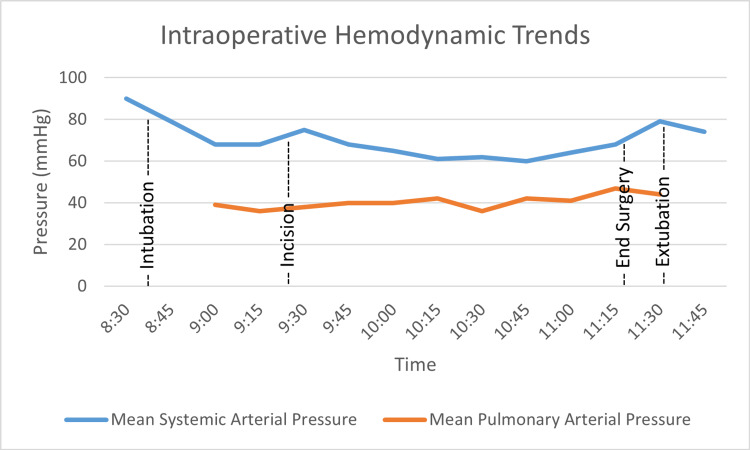
Intraoperative trends in mean systemic arterial pressure and mean pulmonary arterial pressure.

Inhaled nitric oxide at 40 parts per million (ppm) and vasopressin 0.02 units per minute (units/min) were initiated, followed by an inhalational induction with sevoflurane and fentanyl 100 micrograms (mcg) in divided 25 mcg doses. Once the patient became unresponsive with loss of lid reflex, rocuronium 100 milligrams (mg) was administered, and an uneventful rapid sequence intubation was performed. Following intubation, a pulmonary arterial catheter (PAC) and transesophageal echocardiography probe were inserted. Anesthesia was maintained with sevoflurane, titrated to bispectral index (BIS, Aspect Medical Systems, Newton, MA, USA) value of 50-60, and rocuronium dosed as needed to maintain paralysis. Peak end-tidal sevoflurane was 1.1% at the time of induction and was gradually tapered down to 0.0% during closure. Epidural infusion of 0.0625% bupivacaine with 10 mcg/mL hydromorphone was begun at 8 mL per hour after removal of the gall bladder but terminated after 30 minutes due to concern for its contribution to the hemodynamic deterioration described below. No additional opioids were administered. Ventilation was maintained in a pressure-regulated volume control mode, and peripheral oxygen saturation (SpO2) was maintained in the low-to-mid-90s on a fraction of inspired oxygen (FiO2) 0.8 and positive end-expiratory pressure 5 cmH2O. A recruitment maneuver to 20 cmH2O performed between induction and incision and intended to maximize and maintain alveolar patency caused a transient decrease in systemic blood pressure from 110s/60s mmHg to 60s/30s mmHg. At the 90-minute mark of surgery, systemic pressures decreased to 80s/40s mmHg despite the increase in vasopressin infusion to 1.4 units/min and twelve 1-unit vasopressin boluses. Furthermore, IV milrinone at 0.4 mcg/kg/min and nitric oxide continued at 40 ppm; pulmonary pressures increased from the 50s/30s mmHg to within 20% systemic pressures. With epinephrine boluses (2.5 mcg x 5), systemic pressures elevated without widening the systemic to pulmonary gradient. Echocardiography revealed moderately to severely reduced RV fractional area change, indicating a severely reduced RV ejection fraction. The closure was completed two hours after incision, at which time neuromuscular blockade was reversed with sugammadex and pressure support weaned to spontaneous unassisted ventilation with continuous positive airway pressure (CPAP) 5 cmH2O. The estimated blood loss was 100 mL. His hemodynamic profile improved with a widened systemic to pulmonary pressure gradient and satisfactory systemic (90s/50s mmHg) and pulmonary (60s/40s mmHg) pressures. He was extubated to nasal CPAP and transferred to the ICU stable, awake, and comfortable. Intraoperative hemodynamic trends are shown in Figure 1. At the time of a routine postoperative check, approximately one hour later, his systemic pressure was 143/88 mmHg, and pulmonary arterial pressure was 86/57 mmHg.

## Discussion

Increased postoperative complications and mortality rates in PH patients are well described. Lai HC et al. reported a mortality rate of approximately 10% in non-cardiac surgery patients with pulmonary arterial systolic pressures >70 mmHg compared to 0% in case-matched controls [1]. Of 18 million non-cardiac surgery admissions, Smilowitz NR et al. found major adverse cardiac events in 8.3% of PH patients versus 2.0% without [2]. In a prospective study of non-cardiac, non-obstetric surgical patients with relatively well-controlled pulmonary arterial hypertension, Meyer S et al. reported 3.5% overall mortality [3]. Of particular concern for the case at hand, Thangamathesvaran L et al. reported that in laparoscopic procedures, PH conveyed a nearly four-fold increase in the relative risk of postoperative cardiac complications [4]. Successful navigation of the surgical period relies on medical optimization, multidisciplinary care planning including a PH specialist, and the conduct of surgery in settings with experienced surgeons, anesthesiologists, and intensivists. Key anesthetic considerations are provided in Table 1.

**Table 1:** Anesthetic considerations in severe pulmonary hypertension. TEE: Transesophageal echocardiography; PAC: Pulmonary arterial catheter; SBP: Systolic blood pressure; PAP: Pulmonary arterial pressure; SpO2: Peripheral oxygen saturation; PaCO2: Carbon dioxide tension.

Anesthetic considerations in severe pulmonary hypertension
Obtain large bore IV access
Preoperative arterial line is recommended to facilitate rapid identification of potentially reversible hemodynamic compromise
TEE may be employed to provide continuous echocardiographic assessment of heart function and guide therapy
PAC may be employed to provide cardiac output, right heart pressure, and mixed venous oxygen saturation measurements
Maintain SBP within 20% of baseline and PAP/SBP ratio at baseline or below
Closely monitor gas exchange to maintain normal SpO2 and PaCO2
Pulmonary vasodilators, vasopressors, and inodilators should be immediately available
Employ a multimodal pain management plan, prioritizing regional techniques when possible

For PH patients, mechanical ventilation presents a twofold decrement in RV stroke volume and cardiac output with decreased RV preload (from increased intrathoracic pressures and increased RV afterload to increased alveolar pressure) [5]. Increased alveolar pressures can worsen RV ejection in a compromised RV by increasing pulmonary vascular resistance, further impairing left ventricular preload and ejection volume. This can rapidly progress to a downward spiral of decreased systemic pressures, reduced coronary perfusion pressures, and cardiac ischemia. Despite minimal anesthesia and escalating doses of vasopressors and inotropes, our patient's hemodynamic deterioration exemplifies the challenges faced. Laparoscopy can be expected to exacerbate these pathophysiologic effects. Popescu WM et al. observed that pneumoperitoneum in obesity, per se, resulted in new-onset diastolic dysfunction and reduced cardiac output in patients undergoing laparoscopic gastric bypass surgery [6]. Elevated intraperitoneal pressure compromises respiratory function as the resulting shift of the diaphragm cephalad requires delivery of higher peak pressures to achieve pre-pneumoperitoneum tidal volumes. In a prospective study of patients undergoing laparoscopic vs. open gastric bypass surgery, peak inspiratory pressures in the laparoscopy group were approximately 34 cmH2O [7]. The laparoscopic group also experienced higher end-tidal carbon dioxide and carbon dioxide tension (PaCO2), lower pH, higher peak inspiratory pressures, and reduced respiratory compliance. These hemodynamically compromising mechanical effects, together with hypercapnia and acidemia, would be especially damaging in severe PH. Our patient's sensitivity threshold to positive pressure ventilation trespassed during a recruitment maneuver to 20 cmH2O, which resulted in acute hemodynamic decompensation. Leading authors who have published widely on laparoscopy warn that they "do not advocate the use of laparoscopic surgery in morbidly obese patients with severe cardiac, pulmonary, hepatic, or renal dysfunction" [7,8]. The decision on surgical approach takes account of a variety of patient and institutional factors. In this case, weighing the risks involved with pneumoperitoneum drove the preference for open surgery.

## Conclusions

Although minimally invasive technologies facilitate procedures in many patients, clinicians must always consider the risks and benefits of the case at hand. Regarding laparoscopy, there is no explicit agreement at which point along the spectrum of PH disease severity the potential harm of pneumoperitoneum begins to outweigh the expected benefits. In the case of intraabdominal surgery for patients with severe pulmonary hypertension, the risk assessment by the surgical and anesthesia team should consider the possible harms of laparoscopy versus alternative approaches as part of their risk mitigation strategy.
